# How do women prepare for pregnancy in a low-income setting? Prevalence and associated factors

**DOI:** 10.1371/journal.pone.0263877

**Published:** 2022-03-14

**Authors:** Loveness Mwase-Musicha, Michael G. Chipeta, Judith Stephenson, Jennifer A. Hall

**Affiliations:** 1 EGA Institute for Women’s Health, University College London, London, United Kingdom; 2 Ministry of Health, Lilongwe, Malawi; 3 Big Data Institute, University of Oxford, Oxford, United Kingdom; University of Washington, UNITED STATES

## Abstract

**Background:**

Despite growing evidence of pregnancy preparation benefits, there is little knowledge on how women in developing countries prepare for pregnancy and factors influencing their preparedness for pregnancy. Here, we determine how women in Malawi prepare for pregnancy and factors associated with pregnancy preparation.

**Methods:**

We used data from a previous cohort study comprising 4,244 pregnant mothers, recruited between March and December 2013 in Mchinji district, Malawi. Associations of pregnancy preparation with socio-demographic and obstetric factors were tested for using mixed effects ordinal regression, with the likelihood ratio and Wald’s tests used for variable selection and independently testing the associations.

**Results:**

Most mothers (63.9%) did not take any action to prepare for their pregnancies. For those who did (36.1%), eating more healthily (71.9%) and saving money (42.8%) were the most common forms of preparation. Mothers who were married (adjusted odds-ratio (AOR 7.77 (95% CI [5.31, 11.25]) or with no or fewer living children were more likely to prepare for pregnancy (AOR 4.71, 95% CI [2.89,7.61]. Mothers with a period of two to three years (AOR 2.51, 95% CI [1.47, 4.22]) or at least three years (AOR 3.67, 95%CI [2.18, 6.23]) between pregnancies were more likely to prepare for pregnancy than women with first pregnancy or shorter intervals. On the other hand, teenage and older (≥ 35 years old) mothers were less likely to prepare for pregnancy (AOR 0.61, 95%CI [0.47, 0.80]) and AOR 0.49 95%CI [0.33, 0.73], respectively).

**Conclusion:**

While preconception care may not be formally available in Malawi, our study has revealed that over a third of mothers took some action to prepare for pregnancy before conception. Although this leaves around two thirds of women who did not make any form of pregnancy preparation, our findings form a basis for future research and development of a preconception care package that suits the Malawian context.

## Introduction

Preconception health conditions and behaviours contribute to pregnancy outcomes. Pregnancy preparation or preconception care allows women or couples to seek or undertake interventions including biomedical, behavioural and social health before conception occurs to identify and deal with any risk factors [1]. There is growing evidence that such preconception interventions significantly improve pregnancy outcomes [2,3].

Sub-Saharan Africa (SSA) suffers the highest burden of maternal and child mortality in the world and rates remain very high despite global efforts targeting major reductions [4,5]. In low and middle- income settings such as these, preconception care programmes are either weak or non-existent due to lack of resources, policy guidelines and knowledge of the need for preconception care among couples [6]. While the content of a preconception care package may be clear in high income countries, in low- and middle-income countries there is no consensus as to what such a package should entail [7,8], leading to challenges in implementation, monitoring and evaluation of pregnancy preparation initiatives in these settings. It is unsurprising therefore, that there is limited knowledge on how women in low- and middle-income settings, SSA in particular, prepare for pregnancy and the factors that influence pregnancy preparation. The limited available evidence shows that most pregnancies in SSA are not prepared for and preconception care is almost non-existent [9,10]. This is despite the numerous underlying health problems such as high prevalence of HIV, adolescent pregnancies, undernutrition and intimate partner violence, putting women at an increased risk of poor pregnancy outcomes [11–14]. A study in Southern Malawi by Yeatman *et al*. found that most men (60%) and women (73%) did not prepare for pregnancy; only 12% of men and 7% of women were found to have sufficiently prepared for pregnancy, but the preparatory steps taken remain unknown [15]. Lack of pregnancy preparation was prevalent even when the pregnancy was intentional, with 50% of women who had planned to get pregnant reporting to have made no preparations for the pregnancy [16]. A study on folic acid supplementation in Uganda found no woman who had been taking folic acid supplementation at preconception [17]. Furthermore, there is no evidence on whether women in Africa receive preconception counselling prior to getting pregnant [9].

Malawi is a low-income country where maternal mortality rates remain high, estimated at 219.7 per 100,000 livebirths in 2015 [18,19]. Recent studies have shown that more than half of pregnancies in Malawi are unintended suggesting that these pregnancies, and possibly more, are not prepared for [16]. This widespread lack of pregnancy preparation may be contributing to delays in accessing antenatal care in addition to limiting access to interventions dealing with preconception risk factors for adverse pregnancy outcomes. This study aims to understand how women in Malawi prepare for pregnancy and the socio-demographic and obstetric factors that influence pregnancy preparation. This can inform the development and implementation of an effective preconception care strategy that formally includes pregnancy preparation in Malawi’s health care system and is accessible to all women.

## Methods

### Study design and data collection

We used data from a previous prospective cohort study of pregnant mothers in Mchinji, a rural district in central Malawi. The initial study aimed to explore relationships between pregnancy intention and key maternal and neonatal outcomes [16]. Full details of the initial cohort study design and setting are published elsewhere [16]. Briefly, pregnant women were recruited over a period of nine months between March and December 2013 from 25 randomly selected area blocks (out of 49 area blocks of approximately equal population size) of Mchinji district. The twenty-five blocks, which covered about half of the district, were randomly selected and grouped into three zones based on location. Pregnant mothers were identified through key informants, who had village registers and enumerated every household and its members for an ongoing district-wide pneumococcal vaccine surveillance programme. All pregnant mothers from the selected 25 areas were eligible to participate in the study if they were aged 15 years or older and provided informed consent.

The degree of pregnancy intention was measured using the validated Chichewa (Malawi’s local language) version of the London Measure of Unplanned Pregnancy (LMUP) [20,21]. The LMUP is a psychometrically validated measure of the degree of intention of a current or recent pregnancy, consisting of six questions covering contraception use, timing of pregnancy, intention, desire for a baby, discussion with a partner and pre-conception preparation. Responses to each question are scored as zero, one or two. The overall degree of pregnancy intention is measured on a scale of zero to 12 in order of increasing degree of pregnancy intention [22].

Participating mothers were asked all six questions on the LMUP and a further set of demographic and obstetric history questions during pregnancy. The focus of the present study was to carry out a detailed analysis of the participants’ responses to question six of the LMUP, which asks about the mothers’ preconception actions in preparation for their pregnancy, in relation to their demographic and obstetric characteristics (http://www.lmup.com) [20].

### Study variables

#### Dependent and independent variables

The dependent variable was mothers’ preparation for pregnancy. It was measured by the participants’ responses to question six of LMUP. Responses to LMUP question six were summarised into three categories: “No preparation” (if participants did not do any of the actions), “some preparation” (if the participant did any one action) and “prepared” (if they took any two or more actions). The independent variables considered were socio-demographic and obstetric characteristics and previous history of depression (Table 1). In this study, possible episodes of depression before pregnancy were screened by asking pregnant women whether (a) they felt down, depressed or hopeless (low mood) or (b) if they had felt no interest or having little pleasure in doing things (anhedonia) in the year before pregnancy [16]. Affirmative responses to at least one of the questions were put in three categories including: (1) yes to either one or both questions, but episodes only lasted for less than two weeks; (2) yes to either question with episodes lasting for more than two weeks; and (3) yes to both questions and episodes lasting for over two weeks (Table 1).

**Table 1:** List of independent variables with potential to influence pregnancy preparation.

Independent variable	Response Categories
Maternal age at last birthday (years)	≤18; 19–29; 30–34; ≥35
Maternal level of education	None; primary; secondary; tertiary
Marital status	Married; not married
Number of live children	0, 1–3, ≥4
Father’s age at last birthday (years)	<18; 19–29; 30–40; ≥41
Partner’s level of education	None; primary; secondary, tertiary
Socio-economic status	Poorest quantile; next poor quantile; middle quintile; next rich quintile; richest quantile
Distance to closest health facility (km)	<2.5; 2.5–4.99; 5–7.5; ≥7.5
Religion	Christian-Other; Christian-Catholic; Muslim; other religion
Tribe	Chewa; Ngoni; Senga; Yao; other tribe
Birth interval	First birth; within 24 months; 2–3 years; ≥3 years
Depression before pregnancy**	1 or 2, <2 weeks; (2)1, >2 weeks; (3) both

Mothers’ socio-economic status was determined by an asset-based approach whereby data was collected on variables that reflected the mothers’ living standards, including characteristics of their houses, access to utilities and durable assets, such as bicycle or radio owned by their households. These variables were then converted into a single variable of socio-economic status by principal component analysis, which was then divided to group women into the socio-economic quintiles”.

### Data management and statistical analysis

We performed exploratory and descriptive analyses to identify frequencies of respondents, preparedness categories, variable correlations and other background characteristics in relation to the outcome of pregnancy preparation. As the dependent variable was ordinal, we fitted an ordinal regression model for univariate analysis of the association between the dependent variable and each of the independent variables. Likelihood ratio and Wald’s tests were then used to identify independent variables to adjust for and test for their association with pregnancy preparation. We selected all variables that were significant at a 20% significance level for inclusion in the multivariable ordinal model. The final multivariable model included the following socio-demographic and obstetric factors: mother’s age at last birthday, marital status, mother’s education, wealth status, distance to closest health facility, time interval between pregnancies number of live children and history of depression prior to the pregnancy. Both the univariate and multivariable ordinal regressions were run as mixed effects models with geographical cluster included as a random effect. We reported crude and adjusted odds ratios, with their 95% confidence intervals (CI) as measure of uncertainty.

### Ethics approval and consent to participate

Ethical approval, including the approach to include pregnant women aged 15 and over, was provided by the University College London Research Ethics Committee and the College of Medicine Research Ethics Committee at the University of Malawi (approval numbers 3974/001 and P.03/12/1273 respectively).

## Results

All 4,244 mothers who participated in the original cohort study were included in the analysis. The mean age of the mothers was 25 years (range: 15–49). Of the total, 3,905 (92%) of the women were married. A majority 85.7% (n = 3,637) of the 4,244 women, and 71.0% (n = 3,012) of their partners, had not been educated beyond primary level. About 30% (n = 1,235) of the women travelled more than 7.5 kilometres to get to the nearest health facility to access health services. Of the 4,244 women interviewed, 49.3% (n = 2,091) were Christian, followed by 46.8% (n = 1,985) who were Catholics (Table 2). The Chewa tribe was the predominant among the respondents (84.8%; n = 3,587). Close to a third of the women (29.2%; n = 1,240) had not given birth before, while most of them (70.6%; n = 2,980) did not report indications of previous episodes of depression (Table 2). Based on women’s self-reported last menstrual period, pregnancy gestational age at the time of interview ranged from two to nine months (median = 6 months).

**Table 2:** Summary of socio-demographic and obstetric characteristics by level of pregnancy preparation by women in Mchinji.

	Total	Unprepared	Some preparation	Prepared
	N = 4244	n = 2710	n = 1055	n = 469
**Mother’s age**				
< = 18	649 (15.3%)	422 (15.5%)	174 (16.5%)	53 (11.3%)
19–29	2546 (60%)	1538 (56.5%)	674 (63.9%)	334 (71.2%)
30–34	618 (14.6%)	419 (15.4%)	139 (13.2%)	60 (12.8%)
> = 35	431 (10.2%)	341 (12.5%)	68 (6.4%)	22 (4.7%)
**Father’s age**				
< = 18	62 (1.5%)	48 (1.8%)	12 (1.1%)	2 (0.4%)
19–29	2209 (52%)	1326 (48.8%)	609 (57.7%)	274 (58.4%)
30–40	1435 (33.8%)	900 (33.1%)	362 (34.3%)	173 (36.9%)
> = 41	365 (8.6%)	292 (10.7%)	56 (5.3%)	17 (3.6%)
**Marital status**				
Unmarried	339 (8%)	293 (10.8%)	35 (3.3%)	11 (2.3%)
Married	3905 (92%)	2427 (89.2%)	1020 (96.7%)	458 (97.7%)
**Mother’s education**				
None	422 (9.9%)	317 (11.7%)	72 (6.8%)	33 (7%)
Primary	3215 (75.8%)	2081 (76.5%)	814 (77.2%)	320 (68.2%)
Secondary	597 (14.1%)	318 (11.7%)	166 (15.7%)	113 (24.1%)
Tertiary	10 (0.2%)	4 (0.1%)	3 (0.3%)	3 (0.6%)
**Father’s education**				
None	334 (7.9%)	236 (8.7%)	77 (7.3%)	21 (4.5%)
Primary	2678 (63.1%)	1725 (63.4%)	675 (64%)	278 (59.3%)
Secondary	1144 (27%)	699 (25.7%)	289 (27.4%)	156 (33.3%)
Tertiary	18 (0.4%)	6 (0.2%)	4 (0.4%)	8 (1.7%)
**Wealth status**				
Poorest	839 (19.8%)	603 (22.2%)	180 (17.1%)	56 (11.9%)
Second	839 (19.8%)	576 (21.2%)	184 (17.4%)	79 (16.8%)
Middle	838 (19.7%)	547 (20.1%)	224 (21.2%)	67 (14.3%)
Next-rich	839 (19.8%)	503 (18.5%)	229 (21.7%)	107 (22.8%)
Rich	835 (19.7%)	453 (16.7%)	232 (22%)	150 (32%)
Distance**				
<2.5km	576 (13.6%)	356 (13.1%)	119 (11.3%)	101 (21.5%)
2.5–4.99km	994 (23.4%)	637 (23.4%)	247 (23.4%)	110 (23.5%)
5–7.49km	1434 (33.8%)	922 (33.9%)	359 (34%)	153 (32.6%)
>7.5km	1235 (29.1%)	802 (29.5%)	328 (31.1%)	105 (22.4%)
**Religion**				
Christian	2091 (49.3%)	1379 (50.7%)	510 (48.3%)	202 (43.1%)
Catholic	1985 (46.8%)	1233 (45.3%)	504 (47.8%)	248 (52.9%)
Muslim	94 (2.2%)	50 (1.8%)	29 (2.7%)	15 (3.2%)
Other	74 (1.7%)	58 (2.1%)	12 (1.1%)	4 (0.9%)
**Tribe**				
Chewa	3597 (84.8%)	2386 (87.7%)	841 (79.7%)	370 (78.9%)
Senga	207 (4.9%)	57 (2.1%)	115 (10.9%)	35 (7.5%)
Yao	92 (2.2%)	55 (2%)	24 (2.3%)	13 (2.8%)
Ngoni	281 (6.6%)	184 (6.8%)	60 (5.7%)	37 (7.9%)
Other	67 (1.6%)	38 (1.4%)	15 (1.4%)	14 (3%)
**Birth interval**				
First birth	1240 (29.2%)	699 (25.7%)	371 (35.2%)	170 (36.2%)
Within 24 months	1029 (24.2%)	800 (29.4%)	167 (15.8%)	62 (13.2%)
2–3 years	884 (20.8%)	579 (21.3%)	222 (21%)	83 (17.7%)
More than 3 years	1081 (25.5%)	634 (23.3%)	295 (28%)	152 (32.4%)
**Depression**				
None	2980 (70.2%)	1850 (68%)	787 (74.6%)	343 (73.1%)
1 or 2, <2 weeks	651 (15.3%)	420 (15.4%)	155 (14.7%)	76 (16.2%)
One, >2 weeks	537 (12.7%)	398 (14.6%)	99 (9.4%)	40 (8.5%)
Both, >2 weeks	53 (1.2%)	40 (1.5%)	7 (0.7%)	6 (1.3%)
**Live children**				
No children	1352 (31.9%)	759 (27.9%)	399 (37.8%)	194 (41.4%)
1–3 children	2077 (48.9%)	1346 (49.5%)	505 (47.9%)	226 (48.2%)
> = 4 children	815 (19.2%)	615 (22.6%)	151 (14.3%)	49 (10.4%)

**Fig 1:**
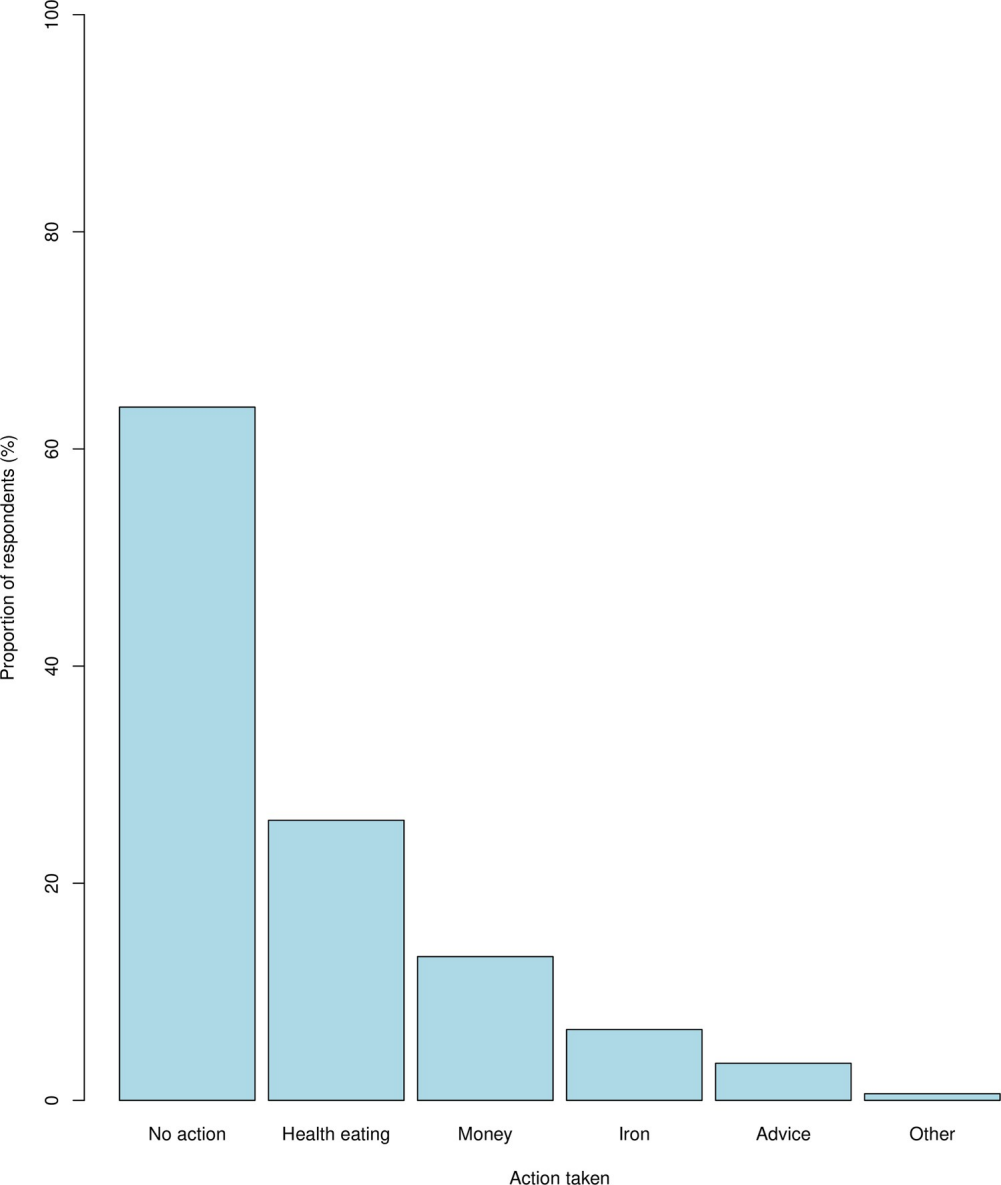
Frequency distribution of actions which pregnant women in Mchinji District, Malawi, took at preconception in preparation for their pregnancy.

Most women (63.9%; n = 2,710) did not take any action to prepare for pregnancy (Fig 1). The most common action taken among those that took some action was healthy eating (n = 1,095) followed by saving money (13.2%; n = 562), taking iron tablets (6.5%; n = 277) and seeking advice (3.4%; n = 145) (Fig 1). A few women (11.1%; n = 469] took a combination of at least two actions (e.g. healthy eating, saving money and taking iron tablets). The most common combination of actions consisted of saving money and eating healthily, which was reported by 8.6% (n = 367) of the women.

After adjustment, pregnancy preparedness was associated with several factors (Table 3). The adjusted odds of preparing for pregnancy were 7.77 (95% CI [5.31, 11.25]) times higher among married women relative to unmarried women. The odds of pregnancy preparation also increased with increasing birth interval. Mothers with a period of two to three years (AOR 2.51, 95% CI [1.47, 4.22]) or more than three years (AOR 3.67, 95%CI [2.18, 6.23]) between current and previous pregnancies were more likely to take preparatory actions for pregnancy as compared to mothers with first pregnancy or with shorter period between pregnancies (Table 3). On the other hand, teenage mothers (AOR 0.61, 95%CI [0.47, 0.80]) and mothers aged above 35 years (AOR 0.49 95%CI [0.33, 0.73]) were less likely to prepare for pregnancy as compared to mothers aged between 19–29 years (Table 3). Women with no live children were also more likely to have prepared for pregnancy (AOR 4.71, 95% CI [2.89,7.61]) than women with 1–3 live children, while mothers with four or more live children were less likely to prepare pregnancy (AOR 0.70, 95%CI [0.53, 0.92]). There was no clear evidence on the role of depression on pregnancy preparation. While mothers who had indicated having low mood or anhedonia for less than two weeks (AOR 0.66, 95%CI [0.53, 0.82]) or just low mood but for more than two weeks (AOR 0.55, 95%CI [0.43, 0.70]) were less likely to prepare for pregnancy as compared to women with no experience of depression, we found no evidence of significant association between pregnancy preparation and episodes of both low mood and anhedonia for more than two weeks (AOR 0.59, [0.28, 1.25]; Table 3), though this analysis is limited by the small number of women who reported this level of depression (n = 53) hence the confidence intervals are wide.

**Table 3:** Factors associated with pregnancy preparation, among women in Mchinji, Malawi.

Variable	Crude OR (95% CI)	Adjusted OR (95% CI)	p (Wald’s test)	p (LR test)
**Marital status: Reference = not married**				**<0.001**
Married	5.96 (4.22, 8.58)	7.77 (5.31, 11.25)	<0.001	
**Mother’s age (years): Reference = 19–29**				**<0.001**
Below 18	0.75 (0.61, 0.92)	0.61 (0.47, 0.80)	<0.001	
30–34	0.71 (0.57, 0.88)	0.87 (0.68, 1.15)	0.368	
35 and above	0.35 (0.26, 0.47)	0.49 (0.33, 0.73)	0.005	
**Mother’s education: Reference = primary**				**<0.001**
None	0.64 (0.49, 0.83)	0.81 (0.61, 1.08)	0.156	
Secondary	1.50 (1.21, 1.85)	1.13 (1.12, 1.43)	0.321	
Tertiary	2.70 (0.64, 12.70)	1.35 (0.29, 6.42)	0.702	
**Wealth status: Reference = poorest**				**0.02**
Second poor	0.98 (0.77, 1.24)	0.84 (0.65, 1.08)	0.170	
Middle rich	1.15 (0.91, 1.45)	0.95 (0.79, 1.22)	0.716	
Next rich	1.28 (1.02, 1.61)	1.13 (0.88, 1.44)	0.340	
Richest	1.35 (1.07, 1.73)	1.16 (0.89,1.52)	0.282	
**Live children: Reference = 1–3**				**<0.001**
None	1.42 (1.21, 1.67)	4.71 (2.89,7.61)	<0.001	
4 and above	0.55 (0.44, 0.68)	0.70 (0.53, 0.92)	0.012	
**Birth Interval: Reference = first birth**				**<0.001**
Below 2yrs	0.35 (0.28, 0.43)	1.02 (0.63, 1.63)	0.944	
2–3 years	0.74 (0.60, 0.91)	2.51 (1.47, 4.22)	0.001	
Over 3 years	0.96 (0.79, 1.17)	3.67 (2.18, 6.23)	<0.001	
**Distance to health facility: Reference = “<2.5 km”**				**0.005**
2.5–5.0km	1.49 (1.11, 1.99)	1.80 (1.32, 2.46)	<0.001	
5.0–7.5 km	1.21 (0.89, 1.64)	1.46 (1.05, 2.03)	0.022	
Over 7.5 km	1.03 (0.74,1.42	1.30 (0.91, 1.82)	0.149	
**Father’s education: Reference = primary**				**0.002**
None	0.87 (0.66,1.15)	1.03 (0.76, 1.49)	0.863	
Secondary	1.27 (1.07,1.50)	1.03 (0.85, 1.25)	0.725	
Tertiary	3.95 (1.29,13.68)	3.19 (0.84, 12.18)	0.091	
**Depression: Reference = none**				**<0.001**
1 or 2, <2 weeks	0.66 (0.53, 0.82)	0.69 (0.55, 0.87)	0.002	
One, >2 weeks	0.55 (0.43, 0.70)	0.66 (0.51, 0.86)	0.002	
Both, >2 weeks	0.48 (0.23 0.95)	0.59 (0.28, 1.25)	0.171	

## Discussion

This study has shown that although pregnancy preparation before conception is not common in Malawi, a sizeable proportion of mothers (just over a third) do take some actions to prepare for pregnancy. This is despite preconception care not being a key component of maternal and child health within Malawi’s health care system, as evidenced by its absence in key Government policy documents such as the 2012 Ministry of Health Road Map for Accelerating Reduction of Maternal and Neonatal Morbidity and Mortality and the 2011–2016 Health Sector Strategic Plan (HSSP). These policy documents largely focus on antenatal care, labour and delivery and the postpartum period [23–25]. The study also revealed that pregnancy preparation is associated with several socio-demographic including age and marital status, and obstetric factors including time interval between pregnancies and number of live children.

Amongst the mothers who had prepared for their pregnancy, the most common ways of pregnancy preparation were eating healthily and saving money, while fewer mothers sought medical attention or took iron. When considered in the global context of how mothers prepare for pregnancy, our findings suggest both similarities and differences in ways of preparing for pregnancy between mothers in high-income countries and mothers in low-income settings. In high-income settings, pregnancy preparation hinges mostly on folic acid intake, changing behaviours or habits such as smoking, alcohol consumption and doing physical exercise and changes in diet to achieve a healthy weight [26]. While the mothers participating in this study also reported diet improvement (eating healthily) as an action they took to prepare for their pregnancy, women in Malawi are generally non-smokers, with less than 1% female smokers, and very few take alcohol especially in rural areas and hence stopping smoking or drinking alcohol is unlikely to be reported among the actions taken to prepare for pregnancy [18]. However, with very high poverty levels, people have irregular income hence the importance of saving money to cover for the financial needs arising from the pregnancy. Also, Malawi has some of the highest anaemia prevalence rates among pregnant women in SSA and indeed globally, ranging between 32.8% and 72.0% [27]. Thus, increasing iron intake from preconception, would help to reduce risks of women developing anaemia during pregnancy [28]. Furthermore, prevalence of HIV and other infectious diseases in Malawi is very high and seeking medical services at preconception would help mothers to test for HIV and other infectious diseases that have potential to bring poor pregnancy outcomes. However, the low proportion of women seeking medical advice at preconception suggests that most mothers may not be aware of their infection status and other non-communicable diseases at conception [18]. This may increase the risk of mother to child transmissions of infections or pregnancy related complications, which may lead to child or maternal morbidity and mortality. The key implication here is that any intervention to improve pregnancy preparation should be in context of the local setting while drawing lessons from the global research and implementation.

Some of the factors identified in this study in association with pregnancy preparation, such as no previous pregnancy or living child or long interval since last pregnancy, imply that pregnancy preparation is linked to a positive intention or desire to be pregnant. Factors such as young age (≤ 18 years) or older age (≥ 35 years) have been associated with poor pregnancy outcomes in other studies [29]. In this study, we have also found that mothers in this age groups were less likely to prepare for pregnancy. The underlying factors for unintended pregnancies included unmet need for, or failure of contraceptives [30]. Considering that these age groups also have some of the highest rates of poor pregnancy outcomes, our findings indicate that interventions to avoid unintended pregnancy through improving access to and uptake of contraception, or to improve pregnancy planning and preparation where pregnancy is wanted in these age groups could be a key to reducing poor pregnancy outcomes.

A limitation of this study is that the data on preconception preparations was collected retrospectively. Interviewing mothers when they were already pregnant on what they did before conception may have introduced recall bias and it is possible some mothers would have been stating what they were doing during pregnancy rather than what they did at preconception. However, the question used to assess preconception preparation was part of a tool that had undergone local translation and validation, suggesting that most women were able to report on actions the took prior to conception [20].

## Conclusion

Although preconception care is lacking in the Malawi health care system, some women are already taking actions to improve their health before conception–principally by eating a healthier diet or saving money. Further research should acknowledge these women’s desire to improve health before pregnancy, and so take a new women-led approach to developing women-led or couple-led interventions to preconception health. Lack of pregnancy preparation is associated with a wide range of socio-demographic and obstetric factors. This suggests a need for multi-faceted interventions, including meeting the contraception needs of those with no intention of getting pregnant.
